# Selbstimage und öffentliches Image des Pflegeberufs: eine quantitative und qualitative Querschnittsstudie

**DOI:** 10.1007/s11553-021-00930-0

**Published:** 2022-03-15

**Authors:** Evelyn Julier-Abgottspon, Sarah Brunner-Pfaffen, Christian Eissler

**Affiliations:** 1 Spitalzentrum Oberwallis/Sozialmedizinisches Zentrum Oberwallis, Überlandstr. 14, 3900 Brig, Schweiz; 2 Bern University of Applied Sciences, Bern, Schweiz

**Keywords:** Prestige, Pflege, Querschnittsstudie, Inhaltsanalyse, Xxx, Prestigious, Nursing, Cross-sectional study, Content analysis, Xxx

## Abstract

**Hintergrund:**

Ein schlechtes Image der Pflege stellt für den Beruf ein großes Problem dar.

**Ziel:**

Diese Erhebungen hatten zum Ziel, empirische Daten in Bezug auf das Selbstimage von diplomierten Pflegefachpersonen in der Schweiz am Beispiel des Oberwallis zu erheben und darzulegen, wie diplomierte Pflegefachpersonen in der Schweiz ihr öffentliches Image einschätzen. Das Selbstimage der diplomierten Pflegefachpersonen sollte dann mit deren Vorstellung des öffentlichen Images verglichen werden. Mögliche Zusammenhänge zwischen soziodemografischen Angaben und der Eigenwahrnehmung des Images sollten untersucht werden.

**Methoden:**

Um das Forschungsvorhaben umzusetzen, wurden ein quantitatives und ein qualitatives Design gewählt. Anhand der Porter Nursing Image Scale und mittels Querschnittsdesign wurden 364 diplomierte Pflegefachpersonen zu deren Image befragt. Zeitgleich wurden zwei Fokusgruppeninterviews mit insgesamt 18 diplomierten Pflegefachpersonen aus dem Akutbereich sowie aus dem stationären und ambulanten Langzeitpflegebereich durchgeführt und inhaltsanalytisch ausgewertet.

**Ergebnisse:**

Diplomierte Pflegefachpersonen im Oberwallis haben ein gutes Selbstimage, schätzen sich selbst aber als wenig einflussreich und als abhängig ein. Deren Auffassung des öffentlichen Images ist im Vergleich zu ihrem Selbstimage deutlich negativer.

**Schlussfolgerung:**

Die Entwicklung von Strategien zur Stärkung des Selbstimages und zur Verbesserung des öffentlichen Images in der Lehre und Praxis sind nötig.

## Abstract (en)

**Background:**

A poor image of nursing is a major problem for the nursing profession.

**Objectives:**

The aim of the present survey is to collect empirical data on the self-image of qualified nursing professionals in Switzerland using the example of Upper Valais and to show how nursing professionals assess their public image in this region. The self-image of nursing professionals is compared with their perception of their public image. Possible connections between sociodemographic data and the self-perception of the image are also investigated.

**Methods:**

To implement the research project, a quantitative and a qualitative design was chosen. Using the Porter Nursing Image Scale and a cross-sectional design, 364 nursing professionals were interviewed about their image. At the same time, two focus group interviews with a total of 18 nurses from the acute care sector as well as from the inpatient and outpatient long-term care sector were carried out and analyzed in terms of content.

**Results:**

Nursing professionals in Upper Valais have a good self-image, but consider themselves to be less influential and largely dependent. Their perception of the public image is clearly more negative compared to their self-image.

**Conclusions:**

The development of strategies to strengthen the self-image, especially regarding their influence and independence, and to improve the public image in teaching and practice is necessary.

„Standing ovation“ – seit der COVID‑19-Pandemie wird Pflegenden applaudiert, um ihnen Wertschätzung entgegenzubringen. Vor der Pandemie jedoch war in Verbindung mit der Pflege eher von geringer Entlohnung und schlechten Arbeitsbedingungen die Rede. Besonders in Bezug auf den Fachkräftemangel stellt ein gutes Image des Pflegeberufes aber einen zentralen Faktor dar.

## Hintergrund und Fragestellung

Über die Jahre hat sich das Image von Pflegefachpersonen zwar modernisiert [19], global wird den Pflegefachpersonen aber immer noch ein stereotypisches und geringschätziges Image zugeschrieben [6, 11]. In einer Schweizer Studie wurde aufgezeigt, dass Pflegefachpersonen Stereotypen, welche anhand von Klischees vorliegen, verinnerlicht haben und teils selbst anwenden, um jemandem ihren Beruf zu erläutern [16]. Die Art und Weise, wie sich Pflegende selbst sehen, wird laut Fletcher (2007; [4]) als Selbstimage bezeichnet. Ein negatives Selbstimage fördert und stärkt die Bildung eines schlechten Images in der Gesellschaft [11].

Ein schlechtes oder stereotypisches Selbstimage stellt für den Pflegeberuf ein großes Problem dar. Um dem drohenden Personalmangel entgegenzuwirken, ist ein gutes Image für die Pflege unabdingbar.

Das Ziel der Studie war, Daten zum gegenwärtigen Selbstimage wie auch einer Einschätzung zum öffentlichen Image von diplomierten Pflegefachpersonen in der Schweiz zu generieren. Zudem sollten institutionsspezifische Unterschiede betrachtet werden. Zusammenhangsanalysen zwischen soziodemografischen Angaben der Teilnehmenden (Ausbildung, Arbeitspensum, Berufserfahrung, Alter und Geschlecht) und deren Eigenwahrnehmung des Images vervollständigten die Untersuchung.

## Methode

Um das Forschungsvorhaben zu realisieren, wurden eine quantitative multizentrische Querschnittsstudie und ein qualitatives Design gewählt.

Eingeschlossen wurden Pflegefachpersonen, die mindestens 18 Jahre alt waren. Die Studienteilnehmenden mussten über ein Pflegediplom verfügen und in einer Oberwalliser Gesundheitsinstitution arbeiten. Sie mussten über genügend Deutschkenntnisse verfügen, um die Umfrage verstehen und ausfüllen zu können.

**Abb. 1:**
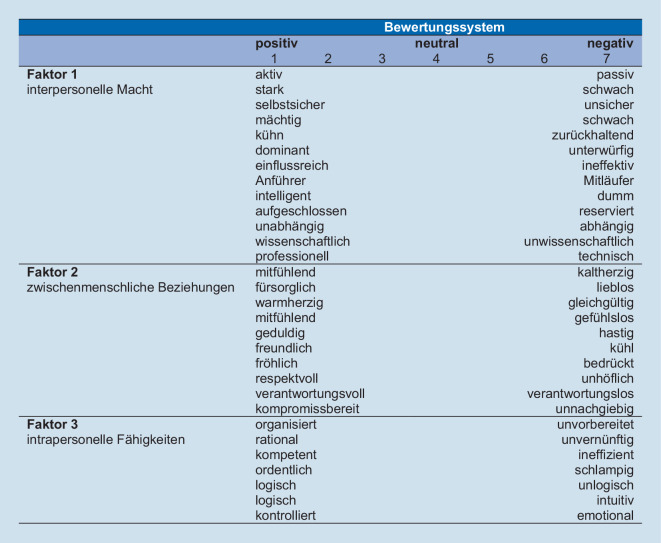
Deutsche Porter Nursing Image Scale (7-stufige Likert-Skala, niedrige Werte bedeuten ein positives Image, hohe ein negatives Image)

Die quantitative Erhebung erfolgte über einen Umfragelink, den alle diplomierten Pflegefachpersonen im Oberwallis erhielten. Es wurde die Umfragesoftware SoSci Survey (SoSci Survey GmbH, Deutschland) genutzt. Neben der Erhebung von soziodemografischen Daten wurde das Image mit der Porter Nursing Image Scale (PNIS) erhoben, welche in englischer Sprache vorlag und in Anlehnung an die Richtlinien von Wild et al. [20] übersetzt wurde (Abb. 1). Die Teilnehmenden wurden angewiesen, die Skala in Bezug auf ihr Selbstimage und darauf zu bewerten, wie sich die Öffentlichkeit Pflegefachpersonen vorstellt. Anhand der siebenstufigen Likert-Skala werden Aussagen zu 30 bipolaren Adjektivpaaren gemacht. Diese werden in drei Faktoren (interpersonelle Macht, zwischenmenschliche Beziehungen, intrapersonelle Fähigkeiten) eingeteilt. Die englische Skala gilt als reliabel und valide [15]. Bei der deutschen PNIS zeigte sich beim Faktor 3 (intrapersonelle Fähigkeiten) eine fragwürdige Reliabilität (Selbstimage Cronbachs α = 0,550; öffentliches Image Cronbachs α = 0,584). Die interne Konsistenz bei beiden Skalen für die restlichen Faktoren ist akzeptabel bis exzellent (Cronbachs α = 0,788–0,904).

Die statistischen Analysen erfolgten mittels Statistical Package for Social Sciences (SPSS, IBM, Stanford, CA, USA). Anhand deskriptiver Statistik wurden das Selbstimage, die Vorstellung des gesellschaftlichen Images sowie soziodemografische Aspekte beschrieben. Unterschiede sowie Zusammenhänge wurden deskriptiv und schließend ergründet. Für die Datenanalyse wurde ein Konfidenzniveau von 95 % gewählt. *P*-Werte *(p)* < 0,05 galten als signifikant. Zur Untersuchung, ob eine Diskrepanz zwischen Selbst- und gesellschaftlichem Image vorliegt, wurden Mittelwertsvergleiche durchgeführt. Der nichtparametrische Kruskal-Wallis-Test wurde verwendet, um institutions-, berufserfahrungs-, ausbildungs-, arbeitspensum- und altersspezifische Unterschiede zu ermitteln.

Um diplomierte Pflegefachpersonen für die Interviews zu rekrutieren, meldeten Pflegedienstleitungen Interessenten. Die ersten 18 wurden im Verhältnis zu den verschiedenen Arbeitsbereichen eingeschlossen. Um die qualitativen Forschungsfragen zu beantworten, wurden zwei Fokusgruppeninterviews mit jeweils 9 Diplomierten durchgeführt. Die Interviews wurden anhand eines semistrukturieren Leitfadens moderiert und zur Datenanalyse digital aufgezeichnet. Der Leitfaden enthielt u. a. Fragen zur Wertschätzung (z. B. „Wie ist die Wertschätzung in eurem Berufsalltag und wie erfahrt ihr diese?“) und zu den Arbeitsbedingungen (z. B. „Welchen Einfluss haben die Arbeitsbedingungen auf das Berufsbild der Pflege?“).

Die Datenanalyse der Interviews erfolgte mittels Codierung und anschließender Kategorisierung. Die Gütekriterien von Miles et al. (2014) wurden berücksichtigt [12].

Für beide Methoden der Datenerhebung wurde ein Pretest durchgeführt.

Die kantonale Ethikkommission hat die Untersuchungen als „nicht bewilligungspflichtig“ beurteilt. Die Studienteilnehmenden wurden schriftlich über die Studie informiert. Die Teilnehmenden unterzeichneten für die Interviews einen „informed consent“. Das Beantworten der Onlineumfrage galt als stille Zustimmung.

## Ergebnisse

Es wurden 667 Pflegefachpersonen zur Teilnahme an der quantitativen Studie eingeladen. Insgesamt konnten die Daten von 364 Personen verwendet werden (Tab. 1).

**Tab. 1:** Merkmale der Studienteilnehmenden (quantitative Studie; *n* = 364)

Merkmale		*n* (%)
Geschlecht	Männlich	31 (8,5)
	Weiblich	332 (91,2)
Alter (Jahre)	≤ 29	55 (15,1)
	30–39	74 (20,3)
	40–49	113 (31,0)
	≥ 50	121 (33,2)
Berufserfahrung (Jahre)	≤ 3	32 (8,8)
	4–10	84 (23,1)
	≥ 11	247 (67,9)
Beschäftigungsgrad (Prozent)	10–40	76 (20,9)
	50–80	141 (38,7)
	90–100	146 (40,1)
Grundausbildung zur Berufszulassung	Diplom HF	88 (24,2)
	Diplom FH	68 (18,7)
	Altrechtlich erworbene Titel	168 (46,2)
	Ausländisches Diplom/anderes	36 (9,9)
Institution	Spitalzentrum Oberwallis	227 (62,4)
	Spitex	67 (18,4)
	Alters- und Pflegeheim/Alterswohnung	60 (16,5)
	Andere	10 (2,8)

*Diplom FH* Diplom Fachhochschule, *Diplom HF* Diplom höhere Fachschule

### Selbstimage/Auffassung des öffentlichen Images

Diplomierte Pflegefachpersonen zeigten tiefe Werte, was auf ein gutes Selbstimage hindeutet. Bei der Auffassung des öffentlichen Images zeichneten sich über alle Faktoren hinweg weniger gute Werte ab. Es zeigte sich stets eine signifikante Diskrepanz zwischen der Einschätzung des Selbstimages und der Ansicht zum gesellschaftlichen Image (Tab. 2).

**Tab. 2:** Unterschiede zwischen dem öffentlichen Image und dem Selbstimage (*n* = 364)

	Öffentliches Image	Selbstimage	Diskrepanz	Gepaarte Differenzen			
				95 %-KI der Differenz			
	*M (SD)*	*M (SD)*	*M (SD)*	Untere	Obere	*T*	*df*
*Faktor 1*	3,27 (0,673)	2,68 (0,565)	0,586 (0,700)	0,514	0,659	15,972^a^	363
*Faktor 2*	2,38 (0,773)	1,71 (0,534)	0,669 (0,698)	0,597	0,741	18,273^a^	363
*Faktor 3*	2,98 (0,604)	2,55 (0,603)	0,422 (0,676)	0,352	0,491	11,894^a^	363
*Summe*	2,76 (0,591)	2,18 (0,437)	0,578 (0,518)	0,524	0,631	21,265^a^	363

7‑stufige Likert-Skala, niedrige Werte bedeuten ein positives Image, hohe ein negatives Image

*df* Anzahl der Freiheitsgrade, *Faktor 1* interpersonelle Macht, *Faktor 2* zwischenmenschliche Beziehungen, *Faktor 3* intrapersonelle Fähigkeiten, *KI* Konfidenzintervall, *M* arithmetisches Mittel, *SD* Standardabweichung, *T* berechnete Differenz

^a^*p* < 0,001

### Institutionelle Unterschiede

Die Resultate für das Selbstimage waren für jeden einzelnen Faktor besser als die Einschätzung in Bezug auf das Image in der Öffentlichkeit, unabhängig von der Institution (*p* = 0,000; Tab. 3).

**Tab. 3:** Institutionelle Unterschiede beim Image

	Öffentliches Image	Selbstimage	Diskrepanz	Gepaarte Differenzen			
				95 %-KI der Differenz			
	*M (SD)*	*M (SD)*	*M (SD)*	Untere	Obere	*T*	*df*
**Spitalzentrum** (*n* = 227)							
*Faktor 1*	3,36 (0,652)	2,70 (0,565)	0,657 (0,678)	0,568	0,746	14,592^a^	226
*Faktor 2*	2,39 (0,764)	1,75 (0,554)	0,645 (0,698)	0,554	0,737	13,932^a^	226
*Faktor 3*	3,02 (0,625)	2,55 (0,603)	0,473 (0,665)	0,386	0,560	10,727^a^	226
*Summe*	2,81 (0,580)	2,20 (0,445)	0,610 (0,517)	0,542	0,678	17,767^a^	226
**Spitex** (*n* = 67)							
*Faktor 1*	3,03 (0,709)	2,64 (0,588)	0,389 (0,778)	0,199	0,579	4,096^a^	66
*Faktor 2*	2,21 (0,704)	1,61 (0,448)	0,601 (0,700)	0,431	0,772	7,032^a^	66
*Faktor 3*	2,94 (0,612)	2,54 (0,625)	0,397 (0,741)	0,216	0,577	4,381^a^	66
*Summe*	2,59 (0,588)	2,12 (0,404)	0,462 (0,549)	0,328	0,596	6,886^a^	66
**Alters- und Pflegeheime sowie Alterswohnungen** (*n* = 60)							
*Faktor 1*	3,22 (0,663)	2,62 (0,570)	0,599 (0,675)	0,424	0,773	6,874^a^	59
*Faktor 2*	2,49 (0,817)	1,67 (0,512)	0,822 (0,684)	0,645	0,998	9,305^a^	59
*Faktor 3*	2,87 (0,520)	2,56 (0,586)	0,312 (0,638)	0,147	0,477	3,786^a^	59
*Summe*	2,75 (0,601)	2,13 (0,414)	0,619 (0,501)	0,489	0,748	9,566^a^	59

7‑stufige Likert-Skala, niedrige Werte bedeuten ein positives Image, hohe ein negatives Image

*df* Anzahl der Freiheitsgrade, *Faktor 1* interpersonelle Macht, *Faktor 2* zwischenmenschliche Beziehungen, *Faktor 3* intrapersonelle Fähigkeiten, *KI* Konfidenzintervall, *M* arithmetisches Mittel, *SD* Standardabweichung, *T* berechnete Differenz

^a^*p* < 0,001

Innerhalb aller Faktoren und bei der Gesamtsumme wiesen Mitarbeitende des Spitalzentrums die am wenigsten positive Ansicht auf. Einzig beim öffentlichen Image bei Faktor 2 (zwischenmenschliche Beziehungen), bei welchem Pflegefachpersonen, die in Alters- und Pflegeheimen arbeiteten, bei der Einschätzung die niedrigsten Werte abgaben (Mittelwert [M] = 2,9; Standardabweichung [SD] = 0,52). Jeder andere Faktor wurde von Pflegefachpersonen der Spitex (ambulante Pflege) in Bezug auf das Selbst- aber auch das öffentliche Image am positivsten angegeben (Tab. 2). Es zeigte sich anhand vom Kruskal-Wallis- und anschließend durchgeführten Post-hoc-Tests, dass Teilnehmende der Spitex das Image in Bezug auf Faktor 1 (interpersonelle Macht; z = 3,930; *p* = 0,000) und die Gesamtsumme der Einschätzung des öffentlichen Images (z = 2,967; *p* = 0,009) signifikant besser einschätzten, als diejenigen des Spitalzentrums.

### Berufserfahrung, Alter, Ausbildungsgrad, Pensum

Gruppenvergleiche in Bezug auf Erfahrung, Alter, Ausbildungs- und Beschäftigungsgrad zeigten keine signifikanten Effekte.

### Geschlecht

Bei den Pflegefachmännern wurde bei jedem Faktor ein negativeres Selbstimage identifiziert als bei Pflegefachfrauen (Tab. 3). Beim zweiten Faktor (zwischenmenschliche Beziehungen) war der Gruppeneffekt signifikant (z = −2,103; *p* = 0,036; Tab. 4).

**Tab. 4:** Selbstimage von Männern und Frauen

Geschlecht	Männlich (*n* = 31)	Weiblich (*n* = 332)		
	Selbstimage	Selbstimage	Mann-Whitney-U-Test	
	*M (SD)*	*M (SD)*	*z*	*p*
*Faktor 1*	2,74 (0,719)	2,67 (0,547)	−0,408	0,683
*Faktor 2*	1,99 (0,749)	1,68 (0,498)	−2,103	0,036
*Faktor 3*	2,63 (0,637)	2,55 (0,601)	−0,622	0,534
*Summe*	2,33 (0,570)	2,17 (0,419)	−1,081	0,280

Niedrige Werte bedeuten ein positives Image, hohe ein negatives Image

*Faktor 1* interpersonelle Macht, *Faktor 2* zwischenmenschliche Beziehungen, *Faktor 3* intrapersonelle Fähigkeiten, *M* arithmetisches Mittel, *SD* Standardabweichung

## Fokusgruppeninterviews

An den Fokusgruppeninterviews haben insgesamt 18 Pflegefachpersonen teilgenommen (Tab. 5).

**Tab. 5:** Merkmale der Studienteilnehmenden (qualitative Studie; *n* = 18)

Merkmale		*n* (%)
Geschlecht	Männlich	2 (11,1)
	Weiblich	16 (88,9)
Alter (Jahre)	20–30	8 (44,4)
	31–65	10 (55,6)
Beschäftigungsgrad (%)	50–80	11 (61,1)
	100	7 (38,9)
Grundausbildung zur Berufszulassung	Diplom FH	9 (50,00)
	Diplom HF	4 (22,2)
	Altrechtlich erworbene Titel	5 (27,8)
Bereich	Akut	6 (33,3)
	Langzeit	6 (33,3)
	Ambulant	6 (33,3)

*Diplom FH* Diplom Fachhochschule, *Diplom HF* Diplom höhere Fachschule

### Wahrnehmung als Pflegefachperson

Während der Gruppendiskussionen zeigte sich, dass durch die Sekundär- und Tertiärausbildungen im Bereich der Pflege, v. a. tertiär ausgebildete Pflegepersonen ihre funktionsspezifischen Tätigkeiten mehr rechtfertigen müssen: „… wenn wir jetzt am Nachmittag zum Beispiel die Pflegedokumentation machen müssen, die doch einen großen Anteil in Anspruch nimmt, […], sagen sie oft: ‚Ja, ihr seid nur am Computer und wir müssen den ganzen Tag rennen.‘ Aber ich glaube, sie sehen nicht, was wir alles machen müssen …“ (1, (1): Gruppendiskussion 1). Die Wahrnehmung durch andere Berufsgruppen wie der Ergo- oder Physiotherapie wurde als gut beschrieben. Die Wahrnehmung wie das medizinische Personal die Pflegefachpersonen sieht, kann in zwei Richtungen beschrieben werden. Die positive Sichtweise sieht so aus, dass Ärzte sehr dankbar für die Pflege sind. Sie sehen Pflegefachpersonen als Teil des multiprofessionellen Teams, wie folgende Äußerung zeigt: „So die Assistenzärzte, die bei uns auf der Abteilung arbeiten, sind teils auch noch sehr froh um die Pflege.“ (1). Es gibt jedoch auch medizinisches Personal, das die Kompetenzen wie auch die Akademisierung der Pflege belächelt und in Frage stellt: „Gerade die Akademisierung der Pflege wird, glaube ich, von den Ärzten, von der Gesellschaft, auch von den eigenen Reihen teils wirklich belächelt …“ (1). Es zeigte sich, dass eine gute Ausbildung und der Einsatz der Fachsprache unabdingbar sind in der Zusammenarbeit mit den Ärzten. Die Fachsprache nimmt eine relevante Rolle ein in Bezug auf die Professionalität des Berufs: „… die Fachsprache, die Argumentation, du musst einfach zeigen, dass du etwas im Kopf hast und nicht einfach die Hilfskraft bist.“ (2, (2): Gruppendiskussion 2). Jedoch zeigte sich hier, dass der Einsatz der Fachsprache, gerade im Langzeitpflegebereich, als Schwierigkeit angesehen wird. Im Team wird der Einsatz der Fachsprache eher als Überheblichkeit betrachtet, um mit dem Wissen andere zu beeindrucken. Die Pflegefachpersonen müssen sich eigenverantwortlich zeigen, für ihre Kompetenzen einstehen und ein kompetentes Auftreten an den Tag legen: „Also ich habe einfach auch gemerkt, du musst einfach wissen, wovon du redest und wenn du kompetent bist auf deinem Gebiet, dann sind sie noch gerne bereit etwas anzunehmen, aber du musst natürlich nicht von irgendetwas quasseln.“ (1).

Das Bild der Pflege in der Gesellschaft wurde ebenfalls sehr zweigeteilt beschrieben. Pflegefachpersonen nehmen wahr, dass ihnen Dankbarkeit und große Wertschätzung für den Pflegeberuf von der Gesellschaft entgegengebracht wird. Auf der anderen Seite wird die Akademisierung der Pflege auch von der Gesellschaft belächelt: „Viele finden das eben unnötig, dass man in der Pflege überhaupt einen Bachelor macht oder dann noch einen Master. ‚Das ist doch absolut unnötig‘ …“ (2).

### Selbstbild – professionelle Identität

Es zeigte sich, dass allgemein eine große Wertschätzung für Pflegefachpersonen, die in anderen Bereichen (Spital, Spitex oder Altersheim) tätig sind, vorliegt. Jedoch wird auch erwähnt, dass Pflegefachpersonen, die in den Langzeitbereich wechseln, häufig von der eigenen Berufsgruppe belächelt werden: „… als ich das kommuniziert habe, hat das niemand begriffen und das ist das, was mich am meisten enttäuscht […] ich getraute mich zeitweise gar nicht zu sagen, wo ich jetzt arbeite.“ (2).

Es wurde auch erwähnt, dass sich die Arbeit der Pflege im Laufe der Jahre stark verändert hat und die administrativen Arbeiten und die Auflagen zugenommen haben: „Die ganzen Auflagen, die hatten wir nicht, ich musste nicht jeden Frigor, jeden Medischrank messen und aufschreiben. Das ist alles nicht gewesen.“ (2).

Die Berufspolitik nimmt einen hohen Stellenwert bei der Beeinflussung des Images ein. Die Akademisierung wird teils begleitet von Vorurteilen und das Studium der Pflege wird aus den eigenen Reihen in Frage gestellt und belächelt. Das wiederum führt dazu, dass sich Pflegestudierende für ihr Studium und ihre Kompetenzen rechtfertigen müssen. Auch Arbeitsbedingungen und Stereotype haben einen großen Einfluss auf das Image: „… du arbeitest ja wirklich mit Nachtwache, mit Pikett, mit Morgenfrüh, Abendspät, zwischendrin drei vier Stunden nichts, Samstag und Sonntag. Es ist sehr, sehr anspruchsvoll […] von dem her ist es nicht übermäßig bezahlt.“ (1).

## Diskussion

Es stellte sich heraus, dass Oberwalliser Pflegefachpersonen ein gutes Selbstimage haben und ihr öffentliches Image gegenüber dem eigenen negativer einschätzen. Die Annahme, dass sich das Selbstimage von Studienteilnehmenden verschiedener Institutionen von deren Einschätzung ihres gesellschaftlichen Images unterscheidet, wurde bestätigt. Die Diskrepanz unterscheidet sich kaum von Resultaten aus Erhebungen anderer internationaler Studien, bei welchen zur Erfassung des Images von Pflegefachpersonen ebenfalls die PNIS oder ein ähnliches Instrument verwendet wurde [11, 15, 17, 18].

### Öffentliches Image

Verglichen mit anderen Studien [1, 6] wurde das öffentliche Image durch die Teilnehmenden dieser Studie besser eingeschätzt. Es wird angenommen, dass Pflegefachpersonen hierzulande vermehrt spüren, dass die Öffentlichkeit sie schätzt. Dass die Pflegefachpersonen das öffentliche Image in Bezug auf interpersonelle Macht (Faktor 1) und intrapersonelle Fähigkeiten (Faktor 3) als am wenigsten positiv einschätzen, unterstützt die Aussage von König (2017; [9]), dass Pflegende zwar für ihre vertrauensvolle Art geschätzt werden, gleichzeitig aber ein begrenztes öffentliches Verständnis ihrer Professionalität und Kompetenz vorherrscht.

Auffallend war, dass Pflegefachpersonen meinen, die Gesellschaft nehme sie als nicht mächtig, abhängig und zurückhaltend wahr. Erklärbar ist dies dadurch, dass die Öffentlichkeit eine Tendenz dazu hat, sie als abhängig von der Ärzteschaft einzuschätzen [19].

Wertschätzung für den Beruf ist vorhanden, jedoch wird sie von Pflegefachpersonen eher als Bewunderung, dass sie die Arbeit machen, wahrgenommen. Die Akademisierung wird nicht als notwendig erachtet. Diese Ergebnisse bestätigen auch die Erkenntnisse von Norman (2015; [14]), welche zeigten, dass andere Berufe wie die Medizin als sehr akademisch angesehen und Pflegefachpersonen weniger intellektuell und akademisch eingestuft werden. Jedoch zeigte sich, dass Pflegefachpersonen selber einen Teil zu dieser Wahrnehmung beitragen. In der Studie von Flaiz (2018; [3]) wurde bestätigt, dass Pflegefachpersonen nach ihrem Studienabschluss ihre Hauptrolle darin sahen, ärztliche Anordnungen auszuführen.

Höchst interessant wäre es, den Einfluss der COVID-19-Pandemie („coronavirus disease 2019“) auf das Selbstimage, v. a. aber auch die Auswirkung auf die Einschätzung des öffentlichen Images durch die Pflegefachpersonen zu überprüfen. Möglich, dass sich die Pandemie global auf das Image von Pflegefachpersonen auswirkt.

### Selbstimage

Oberwalliser Pflegefachpersonen haben ein gutes Selbstimage. Sie denken aber von sich, wenig Macht zu besitzen und nicht sehr unabhängig zu arbeiten. Dies könnte dran liegen, dass im Oberwalliser Gesundheitswesen innovative Versorgungsmodelle, zum jetzigen Zeitpunkt noch wenig etabliert sind. Der zukünftige Einsatz von Advanced Practice Nurses wird die professionsspezifische Identität fördern. Im Rahmen von solch neuen Rollen können sie aufgrund ihrer fachlichen Expertise und Ausbildung selbstständig und unabhängiger arbeiten.

### Institutionelle Unterschiede

Spitex-Mitarbeitende wiesen das positivste Selbstimage aus. Dies passt zur Aussage von De Vliegher et al. (2011; [2]), dass Pflegefachpersonen in der ambulanten Versorgung über ein positiveres Selbstimage verfügen, als solche, die in stationären Einrichtungen arbeiten. Pflegefachpersonen in der Spitex sind bei Hausbesuchen auf sich selbst gestellt. Ein interdisziplinärer Austausch ist aufgrund fehlender räumlicher Nähe erschwert. Es kann davon ausgegangen werden, dass durch die selbstständigere Berufsausübung das Gefühl der Abhängigkeit und der Unterwürfigkeit weniger vorliegt.

### Geschlechtsspezifische Unterschiede

Quantitativ zeigte sich, dass Pflegefachmänner ein negativeres Selbstimage haben. Hochinteressant ist, dass auch beim Faktor 1 (interpersonelle Macht), der u. a. zeigt, wie führungsstark oder einflussreich eine Pflegefachperson die eigene Berufsgruppe einschätzt, eine positivere Ansicht der Frauen festgestellt werden konnte. Diese Erkenntnis kommt stärker zu tragen, wenn bedacht wird, dass etwa 60 % der Männer in der Pflege im Führungsbereich tätig sind [8]. Hinzu kommt die Erwartung, dass bessere Aufstiegs- oder Verdienstmöglichkeiten bei Pflegefachmännern zu einer positiveren Selbsteinschätzung des Images führen würde [6, 7]. Dies wurde nicht bestätigt.

### Einflussfaktoren

Bei der Beeinflussung des Images wird der professionellen Identität ein wichtiger Stellenwert beigemessen. Dazu gehören die Fachsprache, Professionalisierung oder das Berufsverständnis. Es konnte dargelegt werden, dass die Fachsprache unter Pflegenden reduziert eingesetzt wird. Diese sollte, insbesondere in der Kommunikation mit ärztlichem Personal, genutzt werden [10].

Die Professionalisierung wie die Akademisierung der Pflege wird in den Kreisen der Pflegefachpersonen heftig diskutiert [5]. Dies ist teils darauf zurückzuführen, dass sich eine Pflegefachperson früher nicht mit ihrer Ausbildung, sondern mit ihrer Berufung zur Barmherzigkeit auszeichnete [13].

### Limitationen

Es zeigt sich bei der englischen, wie auch deutschen Version der PNIS eine fragwürdige Reliabilität beim Faktor 3. Zudem wurde die Skala 1991 entwickelt und es müssten einige Komponenten überprüft werden.

Qualitativ erfolgten nur zwei Interviews.

## Schlussfolgerungen

Trotz Einschränkungen ist es gelungen, erste Erkenntnisse zum Selbstimage diplomierter Pflegefachpersonen und deren Auffassung des öffentlichen Images zu generieren. Die Ergebnisse lassen darauf schließen, dass sich Pflegefachpersonen bewusst sind, dass ein begrenztes oder verzerrtes öffentliches Verständnis ihrer Professionalität und Kompetenz vorherrscht, dass sie aber ein positives Selbstimage haben.

## Fazit für die Praxis

- Ein positives Selbstimage ist Voraussetzung für ein gutes öffentliches Image der Pflege.
- Ist das Image der Pflege gut, werden sich mehr für eine Ausbildung im Pflegebereich entscheiden und Berufsausübende langfristig im Beruf verweilen.
- Die Entwicklung von Strategien zur Stärkung des Images ist notwendig.
- Pflegefachpersonen sollen sich bewusst sein, dass sich das Selbstimage im gesellschaftlichen Image widerspiegelt und Pflegende als Schlüsselpersonen in Bezug auf die Stärkung des gesellschaftlichen Images gelten.
- Vor allem um das öffentliche Image zu verbessern, aber auch das Selbstimage präventiv weiter zu stärken, müssen Pflegefachpersonen befähigt werden, entsprechende Verhaltensweisen zu entwickeln.
- Ausbildungszentren sollen die berufliche Sozialisation mit einem positiven Selbstwertgefühl fördern, damit ein stereotypisches Image präventiv abgewehrt wird.
